# Supplementary material for: MGL S3 Chimeric Enzyme Drives Apoptotic Death of EGFR-Dependent Cancer Cells through ERK Downregulation
Source: Int J Mol Sci. 2022 Oct 24;23(21):12807. doi: 10.3390/ijms232112807 (PMC9657631; doi:10.3390/ijms232112807)
Supplement: Supplementary file 1 [file ijms-23-12807-s001.zip › ijms-1885114-supplementary.pdf]

Supplementary figures

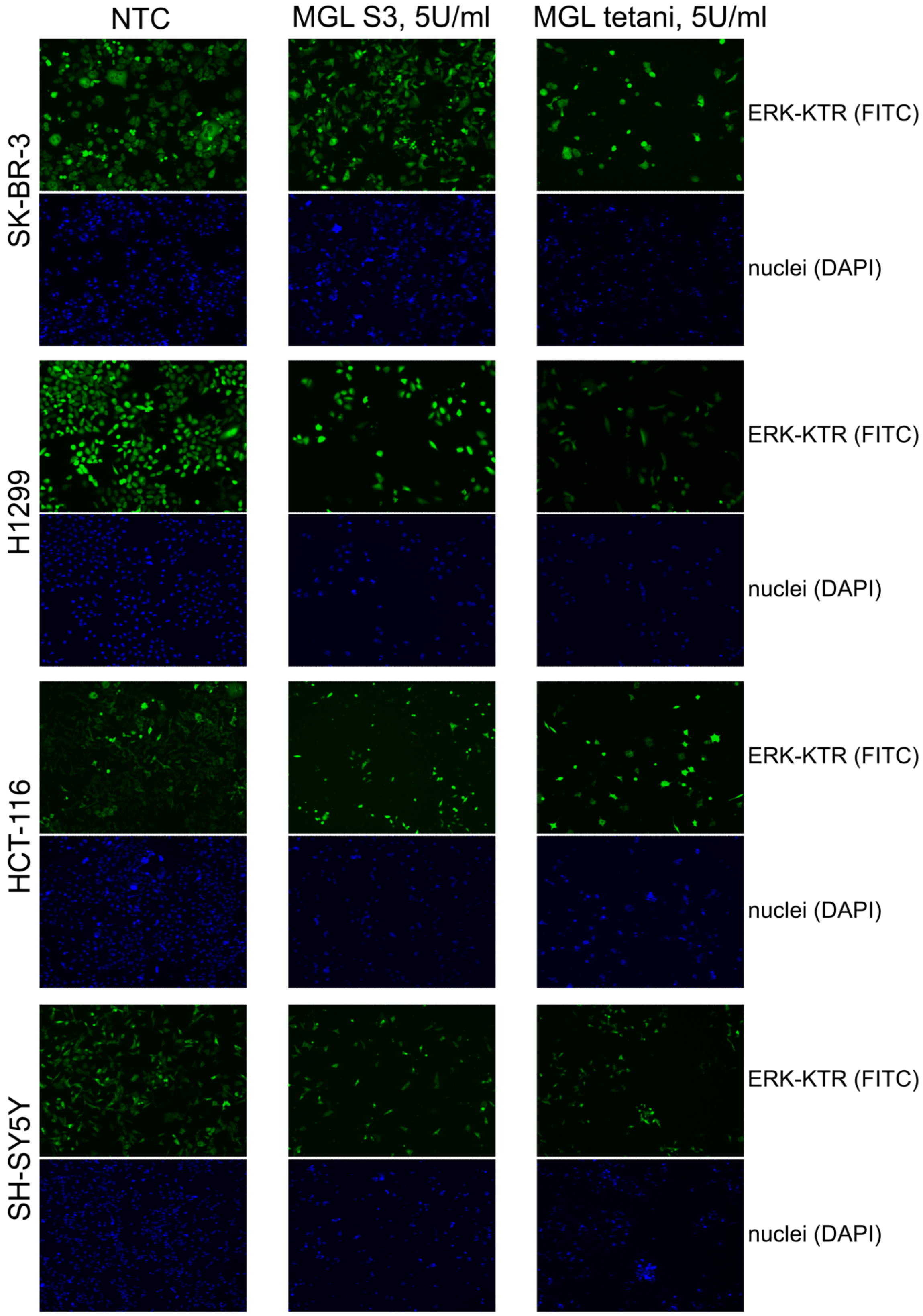

Figure S1. Microphotographs of SK-BR-3, H1299, HCT-116, and SH-SY5Y cells non-treated (NTC) or incubated with 5 U/ml MGL S3 or MGL tetani. Imaging was performed in two

channels – FITC (for ERK-KTR visualization) and DAPI (for Hoechst-33342 staining visualization).

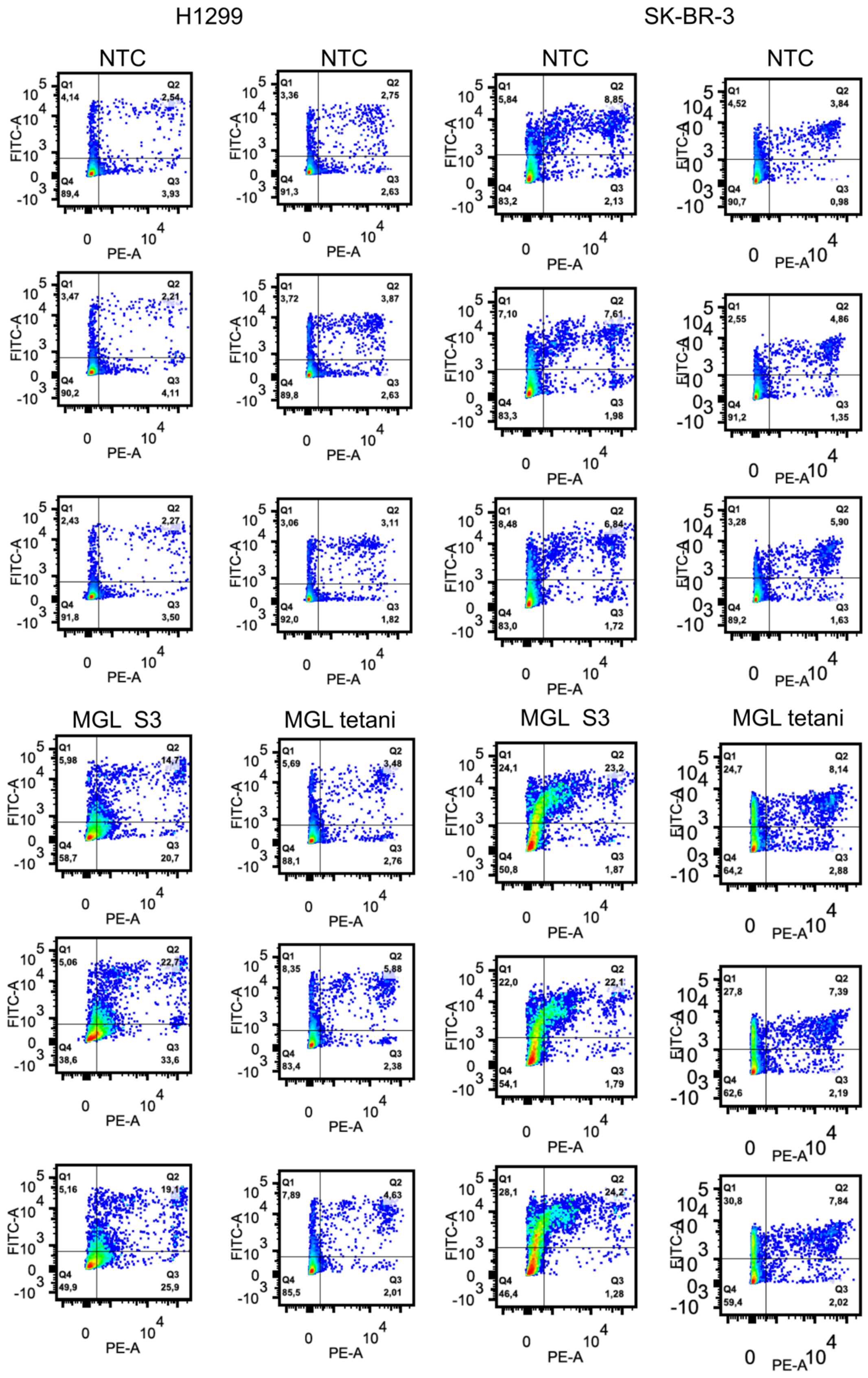

Figure S2. Dot plots representing data acquired by flow cytometry showing the mean fluorescence intensity of H1299 and SK-BR-3 cells stained with Annexin V (FITC-A) and propidium iodide (PE-A). Cells were treated with 2.5 U/ml of MGL S3 or MGL tetani in triplicates.
